# Identification and initial characterisation of a protein involved in *Campylobacter jejuni* cell shape

**DOI:** 10.1016/j.micpath.2017.01.042

**Published:** 2017-03

**Authors:** Diane Esson, Srishti Gupta, David Bailey, Paul Wigley, Amy Wedley, Alison E. Mather, Guillaume Méric, Pietro Mastroeni, Samuel K. Sheppard, Nicholas R. Thomson, Julian Parkhill, Duncan J. Maskell, Graham Christie, Andrew J. Grant

**Affiliations:** a Department of Veterinary Medicine, University of Cambridge, Madingley Road, Cambridge, UK; b Department of Chemical Engineering and Biotechnology, University of Cambridge, New Museums Site, Pembroke Street, Cambridge, UK; c Department of Infection Biology, Institute for Infection and Global Health and School of Veterinary Science, University of Liverpool, Leahurst Campus, Neston, Cheshire, UK; d Wellcome Trust Sanger Institute, Wellcome Trust Genome Campus, Hinxton, Cambridge, UK; e Department of Biology and Biochemistry, University of Bath, Claverton Down, Bath, UK; f The London School of Hygiene and Tropical Medicine, London, UK

**Keywords:** *Campylobacter jejuni*, Cell shape

## Abstract

*Campylobacter jejuni* is the leading cause of bacterial food borne illness. While helical cell shape is considered important for *C. jejuni* pathogenesis, this bacterium is capable of adopting other morphologies. To better understand how helical-shaped *C. jejuni* maintain their shape and thus any associated colonisation, pathogenicity or other advantage, it is first important to identify the genes and proteins involved. So far, two peptidoglycan modifying enzymes Pgp1 and Pgp2 have been shown to be required for *C. jejuni* helical cell shape. We performed a visual screen of ∼2000 transposon mutants of *C. jejuni* for cell shape mutants. Whole genome sequence data of the mutants with altered cell shape, directed mutants, wild type stocks and isolated helical and rod-shaped ‘wild type’ *C. jejuni*, identified a number of different mutations in *pgp1* and *pgp2*, which result in a change in helical to rod bacterial cell shape. We also identified an isolate with a loss of curvature. In this study, we have identified the genomic change in this isolate, and found that targeted deletion of the gene with the change resulted in bacteria with loss of curvature. Helical cell shape was restored by supplying the gene *in trans*. We examined the effect of loss of the gene on bacterial motility, adhesion and invasion of tissue culture cells and chicken colonisation, as well as the effect on the muropeptide profile of the peptidoglycan sacculus. Our work identifies another factor involved in helical cell shape.

## Highlights

• A *C. jejuni* isolate with a loss of curvature was identified.
• A targeted gene deletion of *CJJ81176_1105* in 81–176 and *CJM1_1064* in M1 were created.
• Defined gene deletion mutants of *CJJ81176_1105* and *CJM1_1064* alter *C. jejuni* motility and interaction with Caco-2 cells.
• Defined gene deletion mutant of *CJM1_1064* does not alter *C. jejuni* colonisation of chickens.

## 1 Introduction

Infection by *Campylobacter* spp, especially *Campylobacter jejuni,* is considered to be the most prevalent cause of bacterial diarrhoeal disease worldwide [1]. The bacterium is found in the gastrointestinal tract of healthy animals, especially chickens, destined for human consumption. The helical shape of *C. jejuni* is believed to be important for the bacteria to colonise chickens and during infection, to move through the mucus layer of the gastrointestinal tract and to ‘corkscrew’ into the cells of a human (or other animal) host.

There is limited understanding of how *C. jejuni* adopts a helical morphology. One study identified a mutation in *flhB* that affected flagella formation and apparently correlated with *C. jejuni* becoming rod-shaped [2], but mutations at other sites in the same flagellar gene resulted in bacteria that remained helical. A mutant in *cj1564* (transducer-like protein 3, Tlrp3) has many altered phenotypic characteristics including loss of curvature, but the mechanism for the change in shape is not clear [3]. Occasionally, laboratory strains of *C. jejuni* lose cell curvature and become rod shaped [4]. *C. jejuni* can also undergo a transition from helical cells to rod shaped or coccoid forms in older cultures, and under conditions of stress. It is not clear whether *C. jejuni* can move back and forth between different conformational states during growth. The only genes known to be involved in determination of the helical cell shape of *C. jejuni* are *pgp1* and *pgp2* [5], [6], [7], and their protein products are peptidoglycan (PG) peptidases that are important for PG modification [5], [6].

The bacterial cell wall is important for providing both rigidity and shape to cells and is composed of layers of PG, or murein, which forms the murein sacculus [8]. In Gram-negative bacteria, such as *C. jejuni*, the murein sacculus is very thin and lies in the periplasm between the inner and outer membranes. PG is a web of glycan polymers joined by peptide side chains, which are either directly crosslinked or joined by short peptide bridges. The peptide side chains are synthesised at the inner membrane as pentapeptides and may be cleaved into shorter fragments by a number of peptidases. Peptidases may be endopeptidases or carboxypeptidases depending on whether they cleave an internal or C-terminal amino acid, respectively. Peptidases are also classified by whether they hydrolyse the bond between two d-amino acids (DD) or between a l-amino acid and a d-amino acid (LD or DL). The number and length of peptides attached to the glycan backbone provide unique muropeptide profiles for each bacterium. The PG modification pathway in bacteria is known to contain a wide array of carboxy- and endopeptidases responsible for cleaving monomeric, dimeric and trimeric peptides [9].

To date, only two carboxypeptidases involved in cleaving monomeric peptides have been identified in *C. jejuni*, Pgp1 [5] and Pgp2 [6]. Pgp2 is an ld-carboxypeptidase, which cleaves disaccharide tetrapeptides into tripeptides [6]. Pgp1 is a dl-carboxypeptidase, which cleaves disaccharide tripeptides into dipeptides [5]. Pgp1 activity is metal-dependent and requires the activity of Pgp2 to provide the tripeptide substrate [6]. When either of the *pgp1* or *pgp2* genes is mutated in the laboratory the muropeptide profile radically changes and helical cell shape cannot be maintained [5], [6]. Loss of *pgp1* causes a decrease in dipeptides and tetrapeptides and an increase in tripeptides [5]. Loss of *pgp2* causes a decrease in dipeptides and tripeptides and an increase in tetrapeptides [6]. Furthermore, overexpression of *pgp1* in *C. jejuni* results in a kinked rod morphology, and muropeptide analysis of the *pgp1* overexpressing strain demonstrates a decrease in tripeptides and an increase in dipeptides [5]. Combined, these findings suggest that even subtle changes to proportions of peptides in the PG can affect *C. jejuni* cell shape.

Pgp2 orthologs are present in a wide range of bacteria that display helical, rod, vibroid (curved rod) or coccoid cell shapes [6]. In contrast, Pgp1 is most highly conserved in helical and vibroid species of the Epsilon- and Delta-proteobacteria [5]. The Pgp1 ortholog in *H. pylori*, Csd4, has also been characterised as a necessary determinant of cell shape in this helical pathogen. A defined *csd4* mutant in *H. pylori* generates a rod-shaped strain that exhibits a similar muropeptide profile to Δ*pgp1* in *C. jejuni* [5], [10]. The conserved nature of Pgp1 in particular supports the hypothesis that this protein is fundamental to cell curvature and helical cell shape.

While it is known that peptidases can be redundant [11], [12], single and double knockouts of Pgp1 and Pgp2 do not demonstrate any change to levels of peptide crosslinking [5], [6], suggesting that there remain unidentified PG peptidases in *C. jejuni*. Thus, further identification and characterisation of the enzymes involved in PG synthesis and modification systems and how these enzymes are localised and regulated is required before we can fully understand how helical shape is generated in *C. jejuni*.

We recently performed a visual screen of 1933 transposon (Tn) mutants of *C. jejuni* for changes in cell morphology [13]. Whole genome sequence (WGS) data of the Tn mutants with altered cell shape, directed mutants, wild type (WT) stocks and isolated helical and rod-shaped ‘WT’ *C. jejuni*, identified a number of different genetic mutations in *pgp1* and *pgp2*, which result in a change in helical to rod bacterial cell shape [13]. In addition, we identified an isolate with a loss of curvature. In this study, we report the genome change leading to the loss of curvature and initial characterisation of the gene.

## 2 Materials and methods

### 2.1 Bacterial strains, media and growth conditions

*C. jejuni* strains were routinely cultured on Mueller Hinton (MH) agar (Oxoid) supplemented with 5% defibrinated horse blood (Thermo Scientific) and 5 μg/ml trimethoprim (Tp). Defined mutants and complemented strains were selected on 10 μg/ml chloramphenicol (Cm) or 50 μg/ml kanamycin (Km), as appropriate. *C. jejuni* cultures were grown in standard microaerophilic conditions (5% CO_2_, 5% H_2_, 85% N_2_, 5% O_2_) at 42 °C, unless otherwise indicated. Electrocompetent *Escherichia coli* and *C. jejuni* used in cloning were prepared and transformed as previously described [14]. Bacterial strains and plasmids used in this study are detailed in Table 1.

### 2.2 DNA sequencing

Sanger sequencing was performed by Source BioScience LifeSciences. WGS was performed at the Wellcome Trust Sanger Institute. Isolates were sequenced as multiplex libraries with 100 or 150 base paired-end reads using next-generation Illumina HiSeq^®^ or MiSeq^®^ sequencing technology, respectively. *De novo* draft assemblies were created using Velvet v1.2.08 or v1.2.10 [21] and sequencing reads were mapped to the reference genome using SMALT v.0.6.4 and v.0.7.4 [22]. SNPs and INDELs were called using SAMtools mpileup [23].

### 2.3 Recombinant DNA techniques

Standard methods were used for molecular cloning [24]. Chromosomal and plasmid DNA purification, DNA modification and ligations were performed using commercial kits according to the manufacturers' instructions (QIAGEN, Thermo Scientific, New England Biolabs). DNA concentration was measured using a Nanodrop ND-1000 spectrophotometer (Thermo Scientific). PCR primers were purchased from Sigma (Sigma-Genosys). Thermal cycling was performed in a Gene Amp^®^ PCR System 9700 (PE Applied Biosystems) or T100™ Thermal Cycler (Bio-Rad). Thermal cycling conditions were 96 °C for 2 min, then 30 cycles at 96 °C for 1 min, 55–60 °C for 1 min and 72 °C for 30 s/kb, and finally an extension at 72 °C for 5 min.

### 2.4 Generation of *C. jejuni* defined gene deletion mutants and complemented strains

Targeted gene deletions of *CJJ81176_1105* and *CJM1_1064* were performed by exchanging the gene with a chloramphenicol acetyl-transferase (*cat*) cassette from pRY111 [19]. The *cat* cassette was amplified with primers containing *Pst*I (dare010) or *Sac*I (dare011) restriction endonuclease (RE) target sites. Flanking regions of *CJJ81176_1105* and *CJM1_10643* were amplified using upstream and downstream primers (dare_1001 to 4) containing RE sites matched to the *cat* cassette primers. PCR-amplified fragments were ligated to pUC19 prior to transformation into *E. coli*. Purified plasmid DNA was used to naturally transform *C. jejuni*. The correct genomic rearrangement was confirmed by PCR and sequencing using the primers dare_ck1 and ck2, respectively. Primers used in this study are listed in Table 2.

Complementation of *CJJ81176_1105* and *CJM1_1064* in the targeted deletion strains was performed by amplifying *CJM1_1064* from DNA isolated from WT helical isolates of strain M1 using primers darec_F and darec_R. The PCR product was ligated into the *Campylobacter* shuttle vector pCE107/70 (Km^R^) [18] and transformed into electrocompetent mutants. A novel genetic complementation system that we have developed [20] was used to complement *CJJ81176_1105* in the strain 81-176, since the transformation of *C. jejuni* 81–176 with pCE107/70 was unsuccessful after repeated attempts. DNA isolated from 81 to 176 WT strain served as the template for the amplification of *CJJ81176_1105* coding sequence using primers darec_F and darec_R. The PCR product was cloned into pSV009 (Km^R^) using the *Bam*HI and *Pst*I restriction sites. The resulting plasmid, pSV009-*pgp3c* (Km^R^), was confirmed by PCR and sequencing. Following which, the *CJJ81176_1105* complementation region was amplified by PCR from pSV009-*CJJ81176_1105c* using the primers pSV009_GCampl_FW1/RV1 and subsequently introduced into *C. jejuni* 81–176 by electroporation. Primers used in this study are listed in Table 2.

### 2.5 Muropeptide analysis

PG purification and digestion protocols were adapted from those described in Glauner [25], Li et al. [26] and Frirdich et al. [5]. HPLC of purified and muramidase-digested *C. jejuni* PG was performed in the same manner and using the same instrumentation as described in Christie et al. [27].

### 2.6 Motility assay

The motility of *C. jejuni* was quantified using motility agar made with 0.4%, 0.6%, 0.8% and 1.0% (w/v) select agar (Sigma) in MH broth. Motility agar was used to fill 6-well plates (7 ml of agar per well) 20 min prior to use. *C. jejuni* isolates were transferred *via* pipette tip from 12 h lawn growth (on MH agar plates) into each well of the motility agar. For each strain to be tested, three replicate 6-well plates were incubated for each motility agar concentration. Motility was measured as the diameter of the halo of motility after 12 h incubation.

### 2.7 Culture of Caco-2 cells

Caco-2 cell lines were purchased from the ATCC (CC-L244, HTB-37). Cells were grown using DMEM (Gibco) supplemented with 10% FBS and 1% non-essential amino acids. Cells were routinely grown in 75 cm^2^ tissue culture flasks and incubated at 37 °C with 5% CO_2_ in a humidified atmosphere.

### 2.8 Caco-2 cell infection assays

Caco-2 cells were seeded at 5 × 10^4^ cells on 24 well plates (Greiner) until confluency was observed. Caco-2 cells were infected with different *C. jejuni* strains at a multiplicity of infection (MOI) of 100. To assay adherence/invasion, infected cells were incubated at 37 °C with 5% CO_2_ in a humidified atmosphere for 2 h. At this point, non-adherent bacteria were removed, subjected to 10-fold serial dilutions and plated on BHI blood agar plates with 5 μg/ml trimethoprim. Wells were washed three times with PBS, and cells were lysed with 0.1% Triton-X-100 in PBS for 15 min. Lysed cells were subjected to 10-fold serial dilutions and plated on BHI blood agar plates with 5 μg/ml trimethoprim. To determine the number of internalised bacteria, infected Caco-2 cells were incubated at 37 °C with 5% CO_2_ in a humidified atmosphere. After 2 h, the media overlaying the infected cells was changed to complete DMEM containing 250 μg/ml gentamycin sulphate and infected cells were incubated at 37 °C with 5% CO_2_ in a humidified atmosphere for a further 2 h. Cells were then washed three times with PBS and lysed with 0.1% Triton X-100 in PBS for 10 min. Serial dilutions of the cell lysates were carried out and plated on BHI blood agar plates with 5 μg/ml trimethoprim. Dilutions of mutant *C. jejuni* strains were plated on BHI blood agar plates containing 10 μg/ml chloramphenicol, whereas genetically complemented strains were recovered on MH blood agar plates supplemented with 10 μg/ml chloramphenicol and 50 μg/ml kanamycin. All plates were incubated for 48 h under microaerophilic conditions at 42 °C before colony counting took place. For both total association and invasion experiments, the percentage of *C. jejuni* interacting with Caco-2 cells was calculated as a percentage of the non-adherent fraction, to account for various strain survival within DMEM (n = 3).

### 2.9 Chicken colonisation experiments

All work was conducted in accordance with UK legislation governing experimental animals under project licence 40/3652 and was approved by the University of Liverpool ethical review process prior to the award of the licence.

One-day-old Ross 308 broiler chicks were obtained from a commercial hatchery. Chicks were housed in the University of Liverpool, High Biosecurity Poultry unit. Chicks were maintained in floor pens at UK legislation recommended stocking levels allowing a floor space of 2,000 cm^2^ per bird and were given *ad libitum* access to water and a pelleted laboratory grade vegetable protein-based diet (SDS, Witham, Essex, UK). Chicks were housed in separate groups at a temperature of 30 °C, which was reduced to 20 °C at 3 weeks of age. Prior to experimental infection, all birds were confirmed as *Campylobacter*-free by taking cloacal swabs, which were streaked onto selective blood-free agar (mCCDA) supplemented with *Campylobacter* Enrichment Supplement (SV59; Mast group, Bootle, Merseyside, UK) and grown for 48 h in microaerobic conditions at 41.5 °C. All microbiological media were purchased from Lab M (Heywood, Lancashire, UK).

At 21 days of age birds were infected with 2 × 10^9^ CFU of either *C. jejuni* M1 or the *CJM1_1064* mutant. At 5 days post infection (p.i.), chickens were killed by cervical dislocation. At necroscopy the ceca were removed aseptically and the cecal contents plated onto mCCDA *Campylobacter* selective agar plates for enumeration as previously described [28].

## 3 Results and discussion

### 3.1 Identification of a *C. jejuni* 81–176 isolate with a loss of curvature

**Fig. 1:**
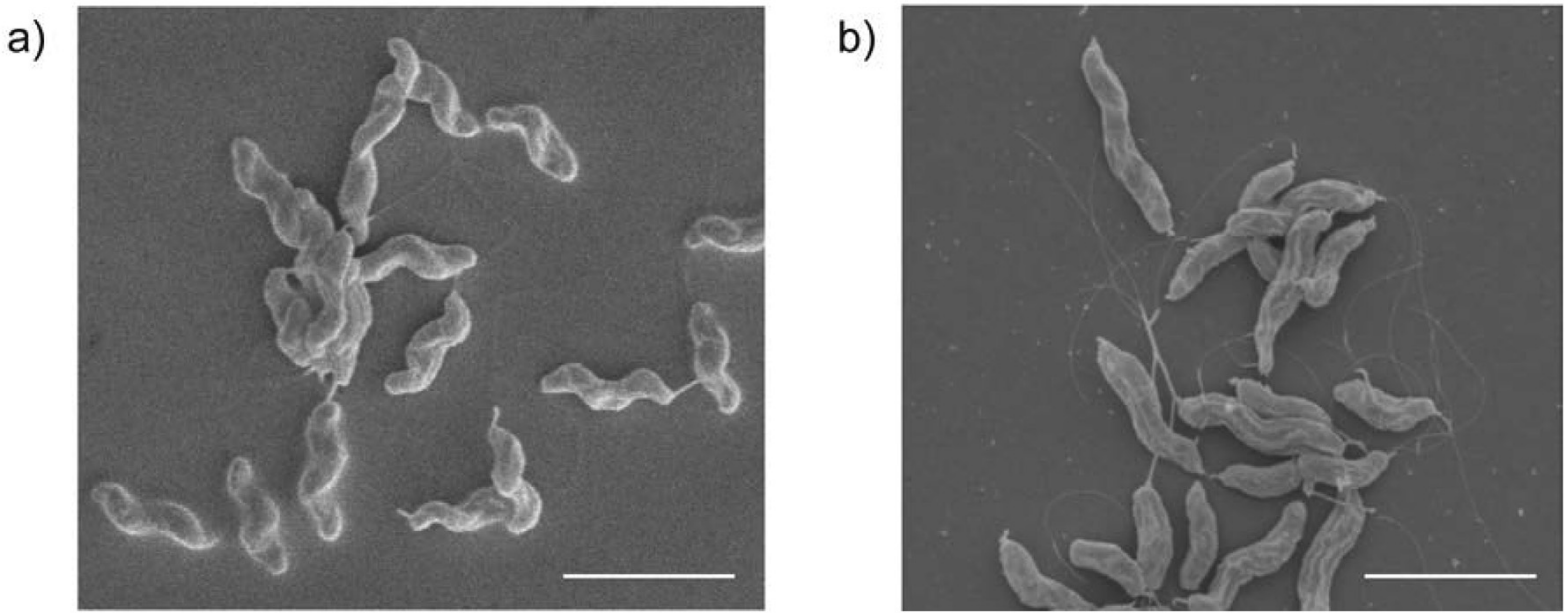
Scanning electron micrographs of helical and loss of curvature morphologies of *C. jejuni* 81–176. (a) 81–176 helical isolate and (b) isolate 81176_KR from the WT *C. jejuni* 81–176 laboratory frozen stock. Scale bars represent 2.5 μm.

When isolating helical and rod bacteria from WT *C. jejuni* strains based on colony morphology as described in Esson et al. [13], we noticed a colony, which was not quite as grey and flat as the typical rod colony morphology but still distinct from the helical colony morphology, and was composed of bacteria with a loss of curvature (‘kinked rod’ cell morphology, 81176_KR). This cell morphology contrasted with the helical morphology of WT 81–176 and was confirmed by scanning electron microscopy (SEM) (Fig. 1). Based on a qualitative assessment of our SEM analyses, there appears to be a difference in the degree of curvature between shorter (presumably younger cells) and longer (presumably older) cells of the ‘kinked rod’ bacteria.

### 3.2 Whole genome sequence analysis of the 81–176 isolate with a loss of curvature

The 81–176 isolate with a loss of curvature (81176_KR) was analysed by WGS and was a change in the number of bases in a documented phase variable region (PVR), and two unique point mutations.

Phase variation (PV) enables genetic and phenotypic variation in a number of bacteria, including *C. jejuni* [29], [30], [31]. Regions of the bacterial genome that are prone to these reversible mutations are called PVR. In *C. jejuni* the PVRs are typically homopolymeric tracts (HTs) that are highly susceptible to slipped-strand mispairings, which alter the length of the tracts and generate frameshift mutations during DNA replication and repair [32], [33]. In this way, PVRs are able to randomly switch genes ‘on’ and ‘off’ and stochastically regulate gene expression [32]. The unique PV pattern was in PVR3 and demonstrated a mostly ‘on’ length, which contrasted with the mostly ‘off’ lengths of other 81–176 isolates. PVR3 in strain 81-176 is 118 bp upstream of *CJJ81176_0590*, encoding a putative uncharacterised protein. This PVR correlates to PVR2 in strain M1, which demonstrated ‘on’ lengths in helical M1 Tn mutants and WT isolates (data not shown). For this reason, as well as the absence of a *CJJ81176_0590* ortholog in strain NCTC11168 [29], we hypothesised that the altered polyG tract upstream of *CJJ81176_0590* was not responsible for the loss of curvature of isolate 81176_KR.

One of the unique point mutations in 81176_KR was a non-synonymous SNP (G > A) in *rpiB* at base location 860819 (CP000538.1). This SNP was predicted to cause a single glutamic acid to lysine amino acid change. The protein product of *rpiB*, ribose 5-phosphate isomerase B, is involved in carbohydrate metabolism [34] and our searches did not demonstrate any link between this enzyme and bacterial cell shape. Therefore, we hypothesised that a single amino acid change to RpiB was not responsible for the observed loss of curvature in 81176_KR.

The other point mutation detected in 81176_KR was an INDEL in *CJJ81176_1105*, a predicted LytM peptidase-encoding gene. This single guanine deletion (2G > G) at base location 1022254 (CP000538.1) was predicted to cause a truncation at residue 65 of the 300 amino acid protein product. BLAST analysis of *CJJ81176_1105* revealed that this gene is highly conserved (>50% coverage and >70% identity) in helical *Campylobacter* spp. (data not shown). We investigated the presence and allelic variances of *CJJ81176_1105* in 859 genomes of *C. jejuni* and *C. coli*. The genomes were from a wide range of isolates: 192 from clinical, agricultural and wild bird sources [35], 319 from multiple stages of poultry processing, including farms, abattoirs and retail chicken meat [36] and 348 from clinical cases [37]. Analysis of these genomes revealed that *CJJ81176_1105* is conserved (data not shown), suggesting this gene is core to both *Campylobacter* species.

### 3.3 Bioinformatic analysis of CJJ81176_1105

A detailed comparison of the translated sequence of *CJJ81176_1105* from four laboratory *C. jejuni* strains (M1, 81116, 81–176 and NCTC11168) demonstrated identical amino acid sequences at all except four residues (Fig. S1). The protein product of *CJJ81176_1105* contains prefoldin, coiled-coil and peptidase domains. Prefoldin is a coiled-coil-containing molecular chaperone that assists in the proper folding of polypeptide products [38]. In eukaryotes, prefoldin is responsible for the folding and localisation of the cytoskeleton components actin and tubulin [39]. The peptidase domain is conserved within the Peptidase M23 (LytM) family, which is composed of zinc-dependent endopeptidases often involved in cell division, elongation and shape determination [40].

Further analysis revealed *CJJ81176_1105* to be orthologous to *csd1* (cell shape determinant 1) in *H. pylori* [41]. In the helical pathogen *H. pylori*, a targeted deletion of *csd1* results in a curved rod morphology, which is fully complemented when *csd1* is supplied elsewhere on the chromosome [41]. Sycuro et al. [41] compared the Csd1 protein product from *H. pylori* to the crystallised LytM endopeptidase from *Staphylococcus aureus*, which demonstrated conserved LytM active site residues in Csd1. Although the *csd1* ortholog in *C. jejuni* was identified by Sycuro et al., the gene was unable to complement the Δ*csd1* phenotype in *H. pylori* [41]. Another group also investigated the role of the *csd1* ortholog in *C. jejuni* morphology but results from these preliminary studies were reported as inconclusive (unpublished work mentioned in Frirdich et al. [5]).

Due to the sequence similarity between *csd1* and *CJJ81176_1105* and the similar ‘intermediate’ morphologies of the *H. pylori* Δ*csd1* strain and 81176_KR, we hypothesised that the frameshift mutation in *CJJ81176_1105* was responsible for loss of curvature morphology of 81176_KR. Moreover, due to its predicted endopeptidase function [41], [42], we hypothesised that the *CJJ81176_1105* protein product might be involved in the same PG modification cascade as Pgp1 and Pgp2.

### 3.4 Defined gene deletion mutants of CJJ81176_1105 alter *C. jejuni* motility and interaction with Caco-2 cells, but not chicken colonisation

**Fig. 2:**
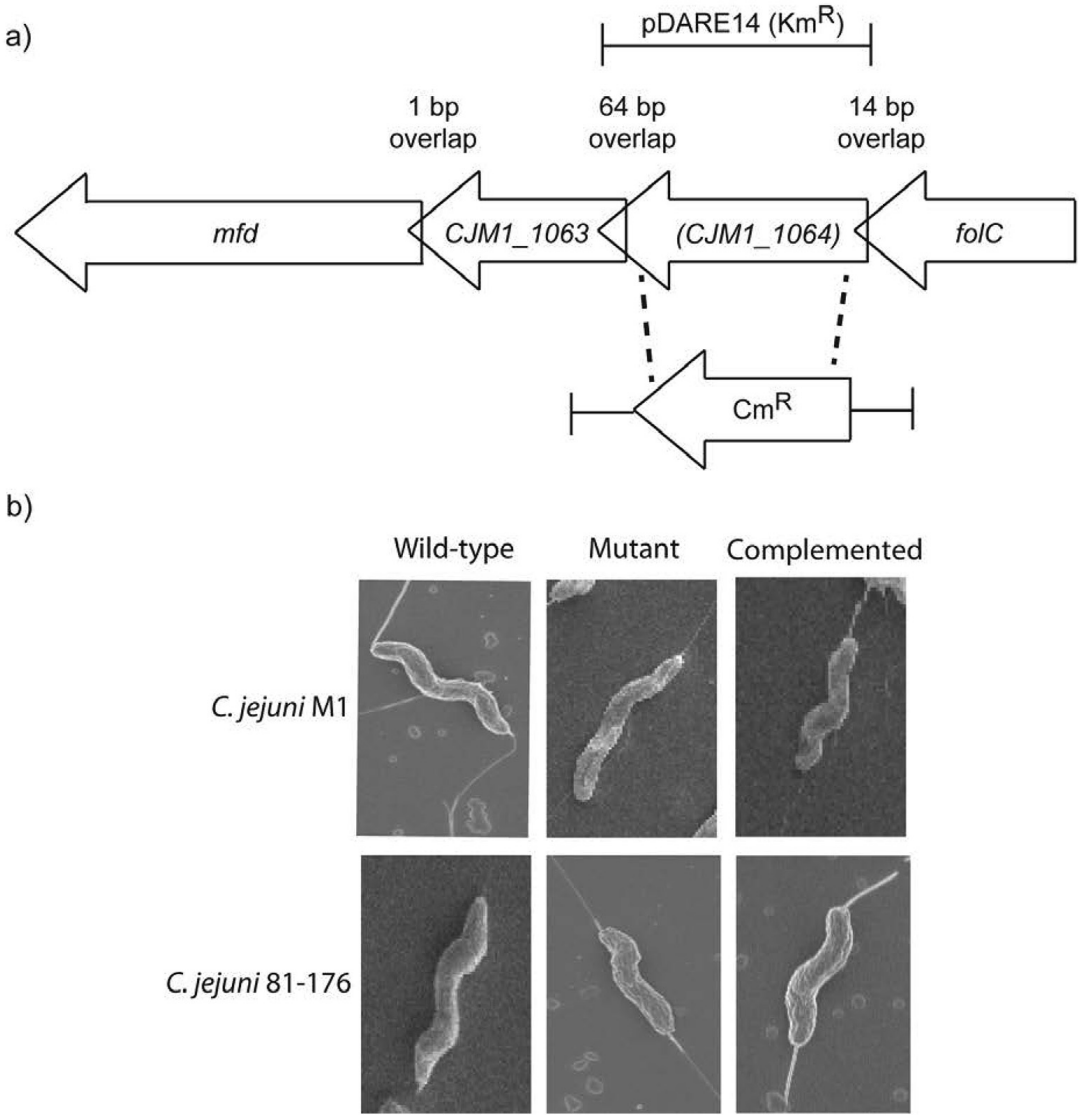
Gene locus and targeted deletion of *CJJ81176_*1105 (81–176) and *CJM1_1064* (M1). (a) A targeted deletion of *CJJ81176_*1105 was generated in the 81–176 and M1 (*CJM1_1064*) backgrounds by exchanging the gene with a *cat* cassette (Cm^R^). The *cat* cassette along with the flanking regions indicated (*CJM1_1064*), was cloned into the suicide vector pUC19 (pDARE10, M1 derivative and pDARE12, 81–176 derivative, Cm^R^). A complementing plasmid (pDARE14, M1 derivative, and pSV009-*CJJ81176_*1105, 81–176 derivative, Km^R^) was generated by cloning M1 *CJM1_1064* into pCE107/70, a kanamycin-resistant shuttle vector. (b) *CJM1_1064* and *CJJ81176_*1105 displayed loss of curvature, complementation with pDARE14 (M1) or pSV009- *CJJ81176_1105c* (81–176) (supplying *CJJ81176_1105 in trans*), strains *CJM1_1064comp* and *CJJ81176_1105comp*, rescued the morphology back to helical.

To test whether the mutation in *CJJ81176_1105* was responsible for the loss of curvature of 81176_KR, we constructed targeted deletions, and complemented strains, on different *C. jejuni* WT backgrounds (*CJJ81176_1105* for strain 81-176 and *CJM1_1064* for strain M1). The defined mutants displayed loss of curvature morphologies, which were restored to helical morphologies by complementation (Fig. 2). From these data, we conclude that *CJJ81176_1105*, and its homolog in other strains, is necessary for a fully helical morphology in *C. jejuni*. We next compared physiological characteristics of the *CJJ81176_1105* and *CJM1_1064* mutants and complemented strains with helical-shaped and rod-shaped WT isolates.

**Fig. 3:**
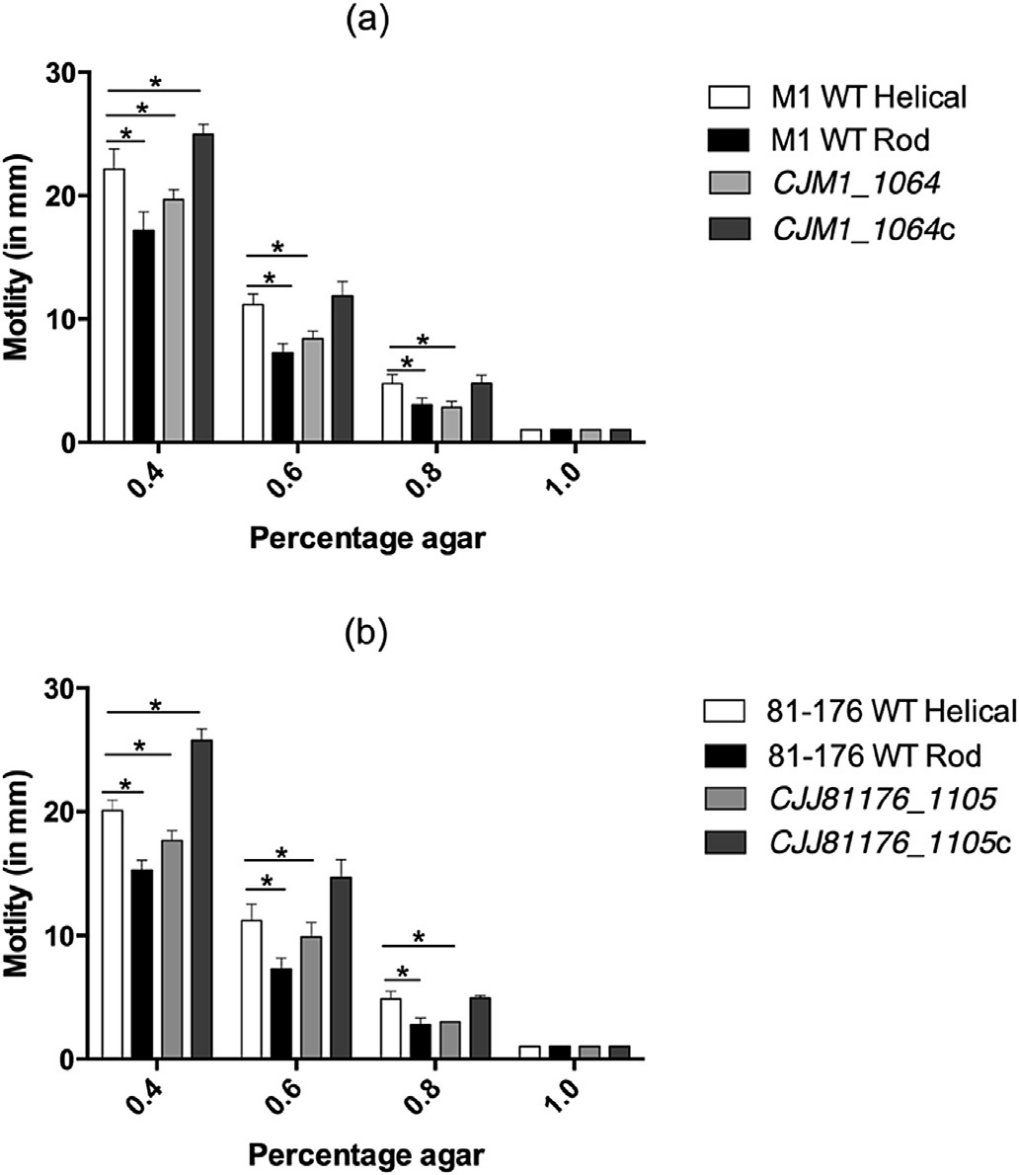
Average motility of helical and rod WT *C. jejuni* M1 isolates, *CJJ81176_*1105 and *CJM1_1064* mutants and complemented strains in 0.4%, 0.6%, 0.8% and 1.0% (w/v) select agar in two different *C. jejuni* backgrounds (a) M1, and (b) 81–176. Statistical differences at each agar concentration were determined using an unpaired t-test correcting for multiple comparisons using a Šídák-Bonferroni method (* = p < 0.005). Data shown is mean and SD (n = 4).

We tested the motility of WT helical and rod isolates, against the *CJJ81176_1105* and *CJM1_1064* mutants and complemented strains, across a range of motility agar concentrations (Fig. 3). The results showed that migration through increasing agar concentrations (decreasing porosity) was significantly reduced in the WT-rod (INDEL in *pgp1*) and *CJJ81176_1105* and *CJM1_1064* mutants compared to the helical isolates (WT-helical and complemented strains). Our work demonstrates that at 0.4 and 0.6% (w/v) agar, the motility of the *CJJ81176_1105* and *CJM1_1064* mutants is slightly greater (although not statistically significant) from the WT-rod isolates. At 0.8% (w/v) agar the motility of the WT-rod (INDEL in *pgp1*) and *CJJ81176_1105* and *CJM1_1064* mutants were comparable, and significantly reduced from the helical isolates. All the isolates were effectively non-motile through 1.0% (w/v) agar, *i.e.* all isolates measured 1 mm in diameter, roughly equivalent to the original pipette stab.

**Fig. 4:**
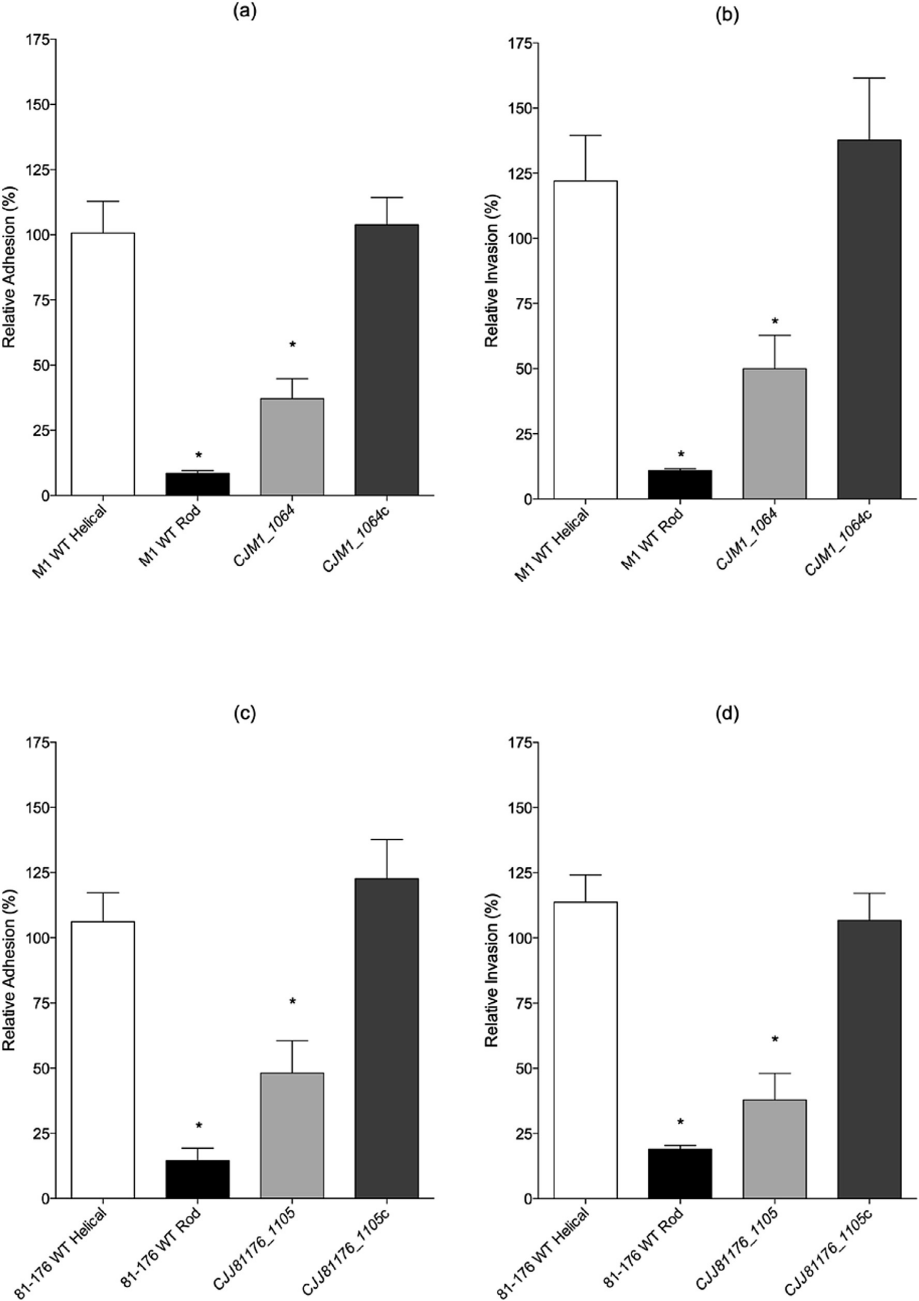
Adhesion (a) to, and invasion (b) of Caco-2 cells by *C. jejuni* M1 and 81–176 rod and helical isolates, *CJJ81176_*1105 and *CJM1_1064* mutants and complemented strains. Data is represented as percentage of wild-type (n ≥ 3) and plotted as means and SEM. Statistical significance was calculated using a Mann-Whitney test where * P < 0.05 and **P < 0.005.

Next, the ability of the *CJJ81176_1105* and *CJM1_1064* mutants to adhere to, and invade, Caco-2 cells was measured (Fig. 4). The WT-rod and *CJJ81176_1105* and *CJM1_1064* mutants displayed statistically significant reductions in adhesion and invasion compared to the WT-helical and complemented strains. For both *C. jejuni* backgrounds, M1 and 81–176, the adherence and invasion of the mutant was slightly greater (although not statistically significant) from the WT-rod isolate (INDEL in *pgp1*).

**Fig. 5:**
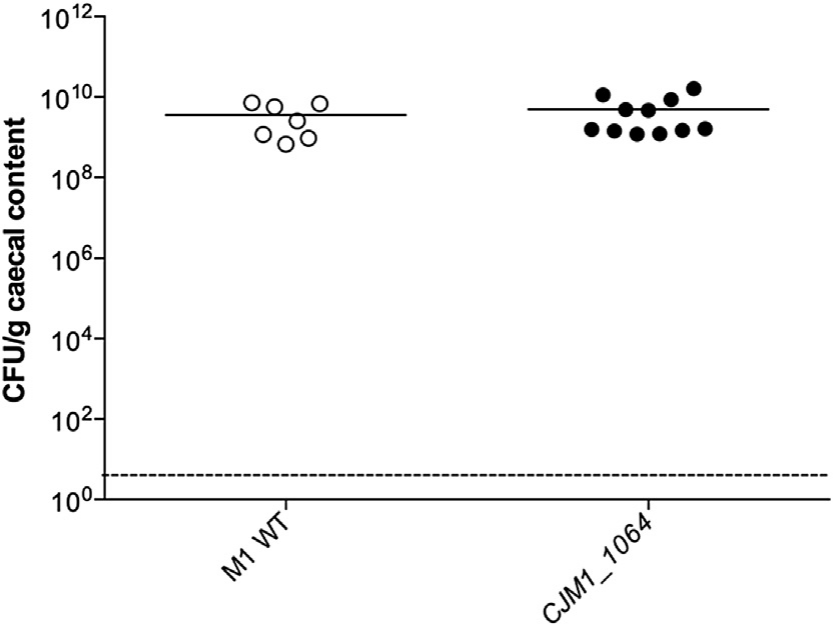
Chicken colonisation of *C. jejuni* M1 WT-helical isolate and *CJM1_1064* mutant. Chickens were orally infected with 0.3 ml of a MH broth culture containing 2 × 10^9^ CFU/ml of the *C. jejuni* isolates. Viable counts from serial dilutions of the cecal contents of chickens show that the WT and mutant colonised to similar levels.

Chicken colonisation experiments were performed using *C. jejuni* strain M1 WT-helical isolate, a natural poultry isolate which is an efficient coloniser of chickens [15], and the *CJM1_1064* mutant. Chickens were inoculated with 2 × 10^9^ CFU of either strain. At 5 days post infection (p.i.), chickens were killed and the cecal contents plated onto mCCDA *Campylobacter* selective agar plates as previously described [28]. The viable counts per gram of cecal contents revealed that there was no difference in the colonisation of the WT or the *CJM1_1064* mutant (Fig. 5).

### 3.5 Muropeptide analysis of helical WT and CJJ81176_1105 (and CJM1_1064) mutant *C. jejuni*

**Fig. 6:**
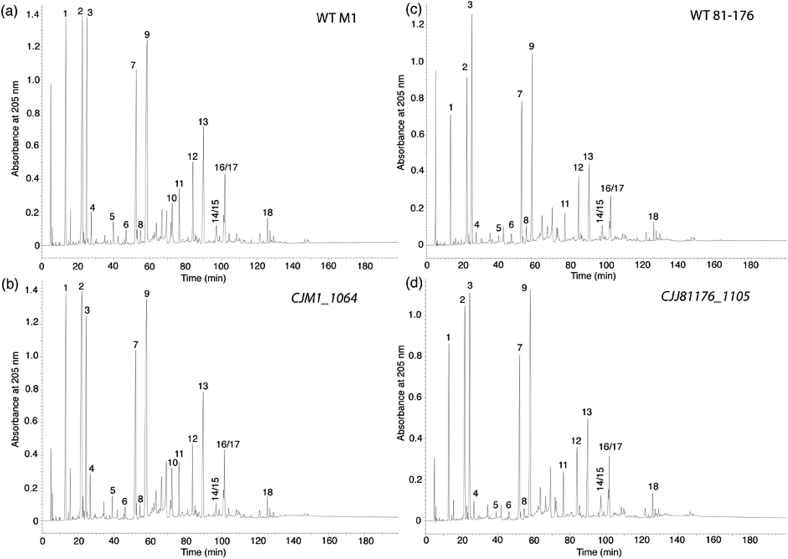
Muropeptide profiles of *C. jejuni* M1 and 81–176 helical WT strains and *CJJ81176_*1105 and *CJM1_1064* mutants. HPLC chromatograms of mutanolysin digested PG purified from *C. jejuni* strain (a) WT M1, (b) M1 *CJM1_1064*, (c) WT 81–176, and (d) 81–176 *CJJ81176_*1105. The muropeptide profiles are very similar between the respective WT and *pgp3* mutant strains. Muropeptide peaks have been putatively numbered and identified according to published muropeptide profiles of strain 81-176 [5].

Muropeptide analysis *via* high-performance liquid chromatography (HPLC) and mass spectrometry (MS) was used to compare the PG sacculi of WT, *CJJ81176_1105* and *CJM1_1064* mutant and complemented *C. jejuni* strains. Muropeptide profiles of mutanolysin-digested PG sacculi isolated from *CJJ81176_1105* and *CJM1_1064* deletion strains appeared virtually identical to muropeptide profiles derived from the parental strains (Fig. 6). Hence in contrast to Pgp1 and Pgp2, *CJJ81176_1105* (and *CJM1_1064*) activity in modelling the PG sacculus appears to be below the threshold for detection *via* the muropeptide analysis technique, and definitive identification of the hydrolytic bond specificity against PG will require further attention.

## 4 Conclusion

There is no effective vaccine against *C. jejuni* and preventative measures aimed at reducing environmental contamination have so far proved ineffective. There is a need for alternative strategies to reduce campylobacteriosis. Most of the *Campylobacteraceae* are helical and it appears that the helical shape of *C. jejuni* is important for its ability to colonise its hosts and cause disease. To address this hypothesis, it is essential to know how helical shape is determined in *C. jejuni*, both genetically and biochemically, but we currently have limited understanding of this. Loss of helical cell shape through interference may hold therapeutic potential by reducing this pathogen's virulence or ability to colonise animals.

This work identifies *CJJ81176_1105* as a novel cell shape determinant in *C. jejuni*. CJJ81176_1105 was identified by the isolation and WGS analysis of a bacterium with loss of curvature within our laboratory WT *C. jejuni* 81-176 stock. This isolate was found to contain a nonsense mutation in *CJJ81176_1105*. Targeted deletions of the gene in both the *C. jejuni* 81–176 and M1 backgrounds reproduced the loss of curvature morphology of the original isolate. This intermediate cell shape was rescued by supplying the gene *in trans*, which confirmed that it is necessary for a fully-helical *C. jejuni* morphology. The homology of CJJ81176_1105 to the endopeptidase Csd1 in *H. pylori* suggests that it may also be involved in this muropeptide cascade.

The intermediate nature of the loss of curvature morphology implies that there exists a hierarchy to helical cell shape maintenance within *C. jejuni*. Based on the evidence demonstrating the importance of endo- and carboxypeptidases in helical cell shape [5], [6], [10], [41], this hierarchy is likely a product of PG peptide lengths and the degree of crosslinking that promotes the cell wall to twist.

As a possible explanation for the differences between the helical, loss of curvature and rod forms of *C. jejuni*, we hypothesise that shorter peptides within the cell wall are localised to the inside of the helix. As such, while the loss of di- and tripeptides in *pgp1* and *pgp2* mutants prevents the maintenance of any curvature [5], [6] the predicted reduction of tetra- and pentapeptide substrates in the *CJJ81176_1105* mutant may merely ration the distribution of di- and tripeptide products throughout the PG, lessening the tension of the helix. To address this hypothesis, future work will require an investigation into the distribution of PG peptides throughout the cell wall *in situ*.

## Funding information

This work was funded by The Wellcome Trust through a PhD training studentship awarded to DE, and was supported by an Isaac Newton Trust/Wellcome Trust ISSF/University of Cambridge joint research grant awarded to AJG. SG was funded by the Biotechnology and Biological Sciences Research Council grant BB/K004514/1. AEM, NRT and JP were supported by the Wellcome Trust grant number 098051. SKS was funded by Biotechnology and Biological Sciences Research Council grant BB/I02464X/1, Medical Research Council grant MR/L015080/1 and Wellcome Trust grant 088786/C/09/Z. GM was supported by a National Institute for Social Care and Health Research Fellowship (HF-14-13). The authors have no conflicting financial interests. The funders had no role in the study design, data collection and interpretation, or the decision to submit the work for publication.

## Figures and Tables

**Table 1:** Bacterial strains and plasmids used in this study.

Strain or plasmid	Relevant genotype or description	Source and/or reference
*C. jejuni* M1	Chicken and human clinical isolate	Diane Newell, [15]
M1 Helical	Helical M1 wild type (M1 isolate, bacteria confirmed to be helical)	This study
M1 Rod	Rod M1 wild type (INDEL in *pgp1*, 8A–7A, leading to a Stop at amino acid 403)	This study
*C. jejuni* 81-176	Human clinical isolate, hyperinvasive	[16]
81176_KR	81-176 wild type with loss of curvature	This study
81-176 Helical	Helical 81–176 wild type (81–176 isolate, bacteria confirmed to be helical)	This study
81-176 Rod	Rod 81–176 wild type (INDEL in *pgp1*, 8A–7A, leading to a Stop at amino acid 403)	This study
*CJJ81176_1105*	Helical 81–176 background, *CJJ81176_1105*, Cm^R^ (loss of curvature)	This study
*CJJ81176_1105comp*	*CJJ81176_1105* background, Cm^R^ Km^R^ (complemented mutant - Helical)	This study
*CJM1_1064*	Helical M1 background, *CJM1_1064*, Cm^R^ (loss of curvature)	This study
*CJM1_1064comp*	*CJM1_1064* background, pDARE14, Cm^R^ Km^R^ (complemented mutant - Helical)	This study
*E. coli* DH5a	Subcloning Efficiency™ DH5α™ Competent Cells. F^−^ Φ80*lac*ZΔM15 Δ(*lac*ZYA-*arg*F) U169 *rec*A1 *end*A1 *hsd*R17(r_k_^−^, m_k_^+^) *pho*A *sup*E44 *thi*-1 *gyr*A96 *rel*A1 λ^-^	Thermo Scientific
**Plasmids**		
pUC19	*E. coli* cloning vector, *C. jejuni* suicide vector, Ap^R^	New England Biolabs, [17]
pCE107/70	*C. jejuni* shuttle vector, Km^R^	[18]
pRY111	Source of *Campylobacter cat* cassette, Cm^R^	[19]
pSV009	*C. jejuni* genetic complementation vector, Ap^R^, Km^R^	[20]
pDARE12	pUC19 derivative encoding *CJM1_1064*, Ap^R^, Cm^R^	This study
pDARE14	pCE107/70 derivative encoding *CJM1_1064*, Km^R^	This study
pSV009-pgp3c	pSV009 derivative encoding *CJ81176_1105*, Ap^R^, Km^R^	This study

Abbreviations for antibiotics: Cm, Chloramphenicol; Km, Kanamycin; Ap, Ampicillin.

**Table 2:** Primer sequences used in this study.

Primer	Target	Sequence (5′ – 3′)
dare008	*cat* cassette	gaattcggtaccCTCGGCGGTGTTCCTTTCCAAGTT
dare009	*cat* cassette	gcatgcggatccCGCCCTTTAGTTCCTAAAGGGTT
dare010	*cat* cassette	gcatgcctgcagCGCCCTTTAGTTCCTAAAGGGTT
dare011	*cat* cassette	agtactgagctcCTCGGCGGTGTTCCTTTCCAAGTT
dare_1001	*CJJ81176_1105* upstream	cccggggaattcAAAAGTGCAGAACGAAAGCTG
dare_1002	*CJJ81176_1105* upstream	tctagagagctcAAAATGTCTTGAACCGTTAATATCTG
dare_1003	*CJJ81176_1105* downstream	gtcgacggatccCCGCATTTGCACTATGAGGT
dare_1004	*CJJ81176_1105* downstream	gttaacgcatgcCCTCAAGTTGCCCTTCAAAA
darec_F	*CJJ81176_1105*	gtcgacggatccGTGGTAAAAAATAAATTCAC
darec_R	*CJJ81176_1105*	agtactctgcagTTACTGTTGTTTCTGAGCTAG
dare_ck1	*CJJ81176_1105*	GGCTATGCTTGATAAATTTCA
dare_ck2	*CJJ81176_1105*	AGTTCCATTAAAGCGACCGCC
pSV009_GCamplif_FW1	Genetic complementation region	TAATAGAAATTTCCCCAAGTCCCA
pSV009_GCamplif_RV1	Genetic complementation region	CTATTGCCATAGTAGCTCTTAGTGG
pSV009_seq_FW1	Sequencing genetic complementation insert	GGAGACATTCCTTCCGTATCT
pSV009_seq_RV1	Sequencing genetic complementation insert	AGCGAGACAAAAACACTGAGC

Upper-case indicates homology to target sequence. Restriction enzyme sites are underlined and preceded by an arbitrary 6-bp sequence.

## Glossary

BHI: brain heart infusion
Cat: chloramphenicol acetyl transferase
CFU: colony forming units
Cm: chloramphenicol
DMEM: Dulbecco's Modified Eagle's Medium
FBS: fetal bovine serum
HPLC: high performance liquid chromatography
INDELs: insertions and deletions
Km: kanamycin
mCCDA: modified charcoal-cefoperazone deoxycholate agar
MH: Mueller Hinton
MOI: multiplicity of infection
MS: mass spectrometry
PBS: phosphate buffered saline
PG: peptidoglycan
p.i.: post infection
PV: phase variable
PVR: phase variable region
RE: restriction enzyme
SEM: scanning electron microscopy
SNPs: single nucleotide polymorphisms
Tn: transposon
Tp: trimethoprim
WGS: whole genome sequence
WT: wild type
w/v: weight per volume
