# Supplementary material for: Identification and initial characterisation of a protein involved in Campylobacter jejuni cell shape
Source: Microb Pathog. 2017 Mar;104:202–11. doi: 10.1016/j.micpath.2017.01.042 (PMC5335918; doi:10.1016/j.micpath.2017.01.042)
Supplement: Online data [file mmc1.docx]

**Supplemental Material**

**
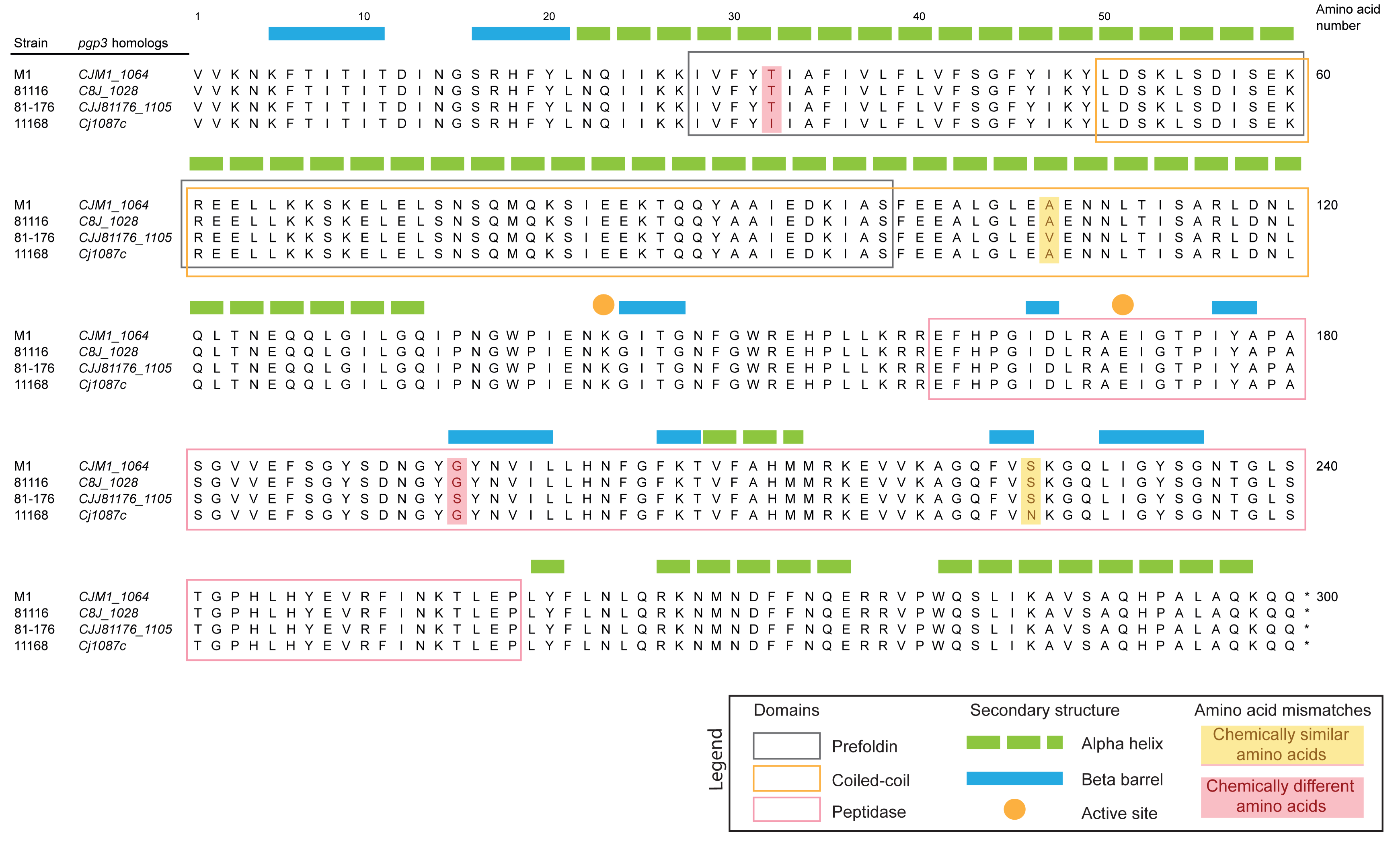
**

**Fig. S1.** Secondary structure and homology of CJJ81176_1105 in four *C. jejuni* strains. The translated sequence of *CJJ81176_1105* in *C. jejuni* M1 (CJM1_1064), 81116 (C8J_1028), 81‑176 (*CJJ81176_1105*) and NCTC11168 (*Cj1087c*) is displayed below the predicted secondary structure of the translated protein (green dashes represent alpha helices; blue bars represent beta barrels). Amino acid differences between the four strains are highlighted by whether side chains are chemically similar (yellow) or distinct (red). Conserved domains are boxed in grey (prefoldin), yellow (coiled-coil) or pink (peptidase). Predicted active site residues are marked with yellow circles. Figure adapted from the secondary structure diagram of *CJJ81176_1105* generated by Phyre2 [S1-S3].

**Supplemental references**

[S1] Altschul SF, Gish W, Miller W, Myers EW, Lipman DJ (1990) Basic local alignment search tool. J Mol Biol 215: 403-410.

[S2] Sycuro LK, Pincus Z, Gutierrez KD, Biboy J, Stern CA, Vollmer W, Salama NR (2010) Peptidoglycan crosslinking relaxation promotes Helicobacter pylori’s helical shape and stomach colonization. Cell 141: 822-833.

[S3] Kelley LA, Sternberg MJE (2009) Protein structure prediction on the Web: a case study using the Phyre server. Nat Protoc 4: 363-371.
